# Prevalence and Burden of Chronic Spontaneous Urticaria in Japan: A Cross-Sectional Study

**DOI:** 10.3390/jcm14041162

**Published:** 2025-02-11

**Authors:** Michihiro Hide, Akihito Uda, Fuyuko Maki, Noriko Miyakawa, Ravneet Kaur Kohli, Shaloo Gupta, Kathryn Krupsky, Bridget Balkaran, Maria-Magdalena Balp

**Affiliations:** 1 Department of Dermatology, Hiroshima Citizens Hospital, Hiroshima 730-8518, Japan; 2 Novartis Pharma K.K., Tokyo 105-6333, Japan; akihito.uda@novartis.com (A.U.); fuyuko.maki@novartis.com (F.M.); noriko.miyakawa@novartis.com (N.M.); 3 Novartis Healthcare Pvt. Ltd., Hyderabad 500081, India; ravneet.kohli@novartis.com; 4 Oracle Life Sciences, Austin, TX 78741, USA; shaloo.gupta@oracle.com (S.G.); kathryn.krupsky@oracle.com (K.K.); balkaranbridget25@gmail.com (B.B.); 5 Novartis Pharma AG, 4056 Basel, Switzerland; maria-magdalena.balp@novartis.com

**Keywords:** anxiety, chronic spontaneous urticaria, depression, prevalence, quality of life

## Abstract

**Background**: Real-world data on the prevalence and burden of patients with chronic spontaneous urticaria (CSU) are limited in Japan. This study aimed to estimate CSU prevalence and assess its humanistic and economic burden. **Methods**: This analysis utilized data from Japanese adult respondents self-reporting physician-diagnosed CSU collected through the 2019 National Health and Wellness Survey. The weighted 12-month prevalence was estimated using 2018 international census projections. Outcomes included the SF-12v2 (physical and mental component summary [PCS and MCS] scores), health utility index (SF-6D and EQ-5D), Dermatology Life Quality Index (DLQI), Generalized Anxiety Disorder-7, Patient Health Questionnaire-9, Work Productivity and Activity Impairment scores at data collection, and healthcare resource utilization over the past 6 months. **Results**: Among 30,006 respondents, 334 reported having CSU, of whom 62.3% were female. The mean (SD) age at data collection and CSU diagnosis was 50.8 (15.3) and 39.2 (14.9) years, respectively. The weighted prevalence of CSU was 1.1%. The mean (SD) PCS and MCS scores were 50.3 (7.0) and 45.1 (10.0), respectively. The mean (SD) health utility measures (SF-6D and EQ-5D) were 0.71 (0.13) and 0.79 (0.18), respectively. The mean (SD) DLQI score was 3.8 (6.0). More than 40% of patients reported mild/moderate/severe anxiety and depression. The mean % (SD) scores for absenteeism, presenteeism, overall work impairment, and activity impairment were 7.6 (17.6), 27.2 (27.2), 30.3 (29.6), and 28.5 (27.8), respectively. Approximately 90.0% of patients visited healthcare providers, including emergency room visits (6.9%) and hospitalizations (9.9%). **Conclusions**: This study provides insights into the diagnosed prevalence and burden of CSU in Japan, highlighting its impact on patients’ lives.

## 1. Introduction

Chronic urticaria (CU) is characterized by the presence of itchy hives and/or angioedema lasting for more than 6 weeks and can be classified into chronic spontaneous urticaria (CSU) or chronic inducible urticaria (CIndU) [1]. CSU presents without a known external cause and is the most common subtype, accounting for approximately two-thirds of all CU cases [2]. The pathophysiology of CSU involves the activation of mast cells and basophils, leading to the release of inflammatory mediators, such as histamine, cytokines, and leukotrienes. This activation may be triggered by autoimmune mechanisms or other underlying immunological dysregulations [1].

The global prevalence of CSU ranges from 0.1% in the United States of America (USA) to 2.7% in China [3,4,5,6,7]. A systematic review with meta-analysis reported a higher prevalence of CSU among women compared to men (1.3% vs. 0.8%), and the study reported a higher prevalence of CSU in the pediatric population (0–19 years) compared to adults (1.4% vs. 0.9%) [8].

CSU adversely impacts the health-related quality of life (HRQoL), particularly when it is associated with angioedema and/or CIndU [2,9]. The presence of comorbidities, such as depression and anxiety among patients with CSU, further impairs the HRQoL of patients [10]. Severe itchiness and the unpredictable nature of hives and angioedema can disrupt sleep and daily activities, negatively affecting family and social interactions [9,11]. CSU also imposes a significant economic burden, driven by high medical costs and treatment-related direct and indirect costs to patients and society [2,10,11].

In Japan, approximately 495,000 patients were diagnosed with CSU in a survey conducted by the Japanese Ministry of Health, Labour and Welfare in 2020 [12]. CSU is also the most prevalent (>66.0%) urticaria subtype in the Japanese population [13].

Among pruritic skin diseases in Japan, urticaria is one of the leading causes of impairments in overall classroom productivity and work productivity and activity [14,15]. CSU is also associated with a higher economic burden in Japanese patients, and the cost of medications can reach up to Japanese yen (JPY) 104,522 per month among patients who are inadequately controlled with antihistamines and step 2 supplemental medications, such as antileukotrienes or histamine H2 blockers [16].

Published evidence on the epidemiology and impact of urticaria, specifically CSU, on patients’ lives in Japan remains scarce. The objective of this study was to assess the prevalence of diagnosed CSU and evaluate the associated humanistic and economic burden in Japan.

## 2. Materials and Methods

### 2.1. Study Design, Data Source, and Patient Population

This noninterventional, retrospective cross-sectional study used data from the 2019 Japan National Health and Wellness Survey (NHWS). The NHWS is a validated, self-reported, internet-based survey reflecting the health and overall disease burden in the general population (aged ≥ 18 years) for more than 200 disease conditions in 12 countries, including Japan [17,18]. Participants were identified via opt-in online survey panels, using quota-sampling procedures to ensure representation of Japan’s general adult population in terms of age and sex [17]. Respondents who experienced CSU in the past 12 months and received a physician diagnosis of CSU were included in this study.

### 2.2. Sociodemographic and Clinical Variables

The variables that were evaluated included sociodemographic (e.g., sex, age, income, employment status, and insurance type) and general health (e.g., body mass index, age at CSU diagnosis, angioedema, diagnosed comorbidities, and treatments) characteristics. The Charlson comorbidity index (CCI) was calculated (0, 1, and 2+ comorbidities), with higher scores indicating worse long-term survival due to comorbid conditions [19,20]. Additionally, patients reported the diagnostician/specialty (dermatologist, primary care physician, and allergist) involved in the diagnosis of CSU.

#### 2.2.1. Measures Used to Assess the Humanistic Burden

All respondents completed the Short-Form 12-Item Survey Version 2 (SF-12v2) questionnaire, a validated patient-reported instrument consisting of 12 questions with a recall period of 4 weeks [21,22]. Responses to the SF-12v2 were summarized by calculating the physical component summary (PCS) and mental component summary (MCS) scores [21]. PCS and MCS scores range from 0 to 100, with higher scores indicating better physical and mental health functioning [23]. The mean (standard deviation [SD]) PCS and MCS scores using a norm-based algorithm were set to 50 (10) for the general population, which were used for interpretation and comparison [22].

The Short-Form 6-Dimension (SF-6D) utility score was generated using SF-12v2 data, with scores ranging from 0 to 1 and higher scores reflecting better health status [24,25]. The EuroQol 5-Dimension (EQ-5D) utility score was calculated using the EuroQol 5-Dimensions 5-Levels (EQ-5D-5L) questionnaire, with scores ranging from 0 to 1, and higher scores reflecting better health status [25]. Respondents rated their health status at the time of the survey on the EuroQol Visual Analog Scale (EQ-VAS), scoring from 0 (the worst imaginable health state) to 100 (the best imaginable health state) [26].

The Dermatology Life Quality Index (DLQI), a 10-item patient-reported questionnaire with a recall period of 7 days, was used to evaluate the dermatology-specific quality of life (QoL) [27]. The DLQI score ranges from 0 to 30, with higher scores representing a greater impact on patients’ lives. Based on the effect on patients’ lives, the DLQI scores can be categorized as no effect (0–1), small effect (2–5), moderate effect (6–10), very large effect (11–20), and extremely large effect (21–30) [27]. In this study, the DLQI scores were also dichotomized, and scores >10 suggest a very large to extremely large effect on patients’ lives and are used as criteria to consider treatment escalations [28].

The Generalized Anxiety Disorder-7 (GAD-7), a seven-item patient-reported measure with a recall period of 2 weeks and a score range from 0 to 21, was used to assess the severity of anxiety [29]. The Patient Health Questionnaire-9 (PHQ-9), with a 2-week recall period of 2 weeks and scores ranging from 0 to 27, was used to assess the severity of depression [30]. Higher scores on both the GAD-7 and PHQ-9 indicate greater severity of anxiety and depression, respectively [31]. The GAD-7 and PHQ-9 scores were dichotomized into scores <5, indicating “none/minimal”, and scores ≥ 5, indicating “mild/moderate/severe” anxiety and depression, respectively [29,30,31,32].

All respondents also completed the 4-item urticaria control test (UCT), with a recall period of 4 weeks, to assess disease control. The UCT scores range from 0 to 16, with a score <12 indicating poorly controlled urticaria [33].

#### 2.2.2. Measures Used to Assess Economic Burden

The Work Productivity and Activity Impairment (WPAI) questionnaire, a six-item patient-reported outcome measure with a 7-day recall period, was used to quantify the impact of CSU on work and activities [34]. Four summary scores were calculated and reported as percent absenteeism, presenteeism, overall work impairment, and activity impairment, with higher scores reflecting greater impairment [34,35]. Absenteeism, presenteeism, and work impairment were reported for employed respondents, whereas activity impairment was reported for all respondents [35].

Respondents also reported the frequency of healthcare provider (HCP) or emergency room (ER) visits and hospitalizations within the past 6 months to evaluate the healthcare resource utilization (HRU). HRU was reported as the percentage of patients using specific resources and the mean (SD) number of visits per patient. Patients reported the HCP specialties visited, including general/family practitioners, allergists, dermatologists, psychologists, and psychiatrists. Respondents were also asked to report their average monthly out-of-pocket expenses.

### 2.3. Statistical Analysis

The 12-month weighted prevalence of diagnosed CSU was calculated. Survey weights were constructed to account for the distribution of sex and age in Japan using the 2018 international census projections [36]. Mean and standard deviation (SD) for continuous variables and count and percentage for categorical variables were used to characterize patient profiles and comorbidities associated with CSU. All the statistical analyses were conducted using SPSS version 23.0 and SAS version 9.4.

### 2.4. Ethical Considerations

Pearl IRB, an independent institutional review board (Indianapolis, IN, USA), examined the study protocol and approved the exemption. The study conformed to the ethical principles stated in the Declaration of Helsinki of 1964. Informed consent was acquired from all respondents for participation in this study, ensuring that patient anonymity was maintained through ethics committee-approved methods.

## 3. Results

### 3.1. Prevalence of Diagnosed CSU

**Figure 1:**
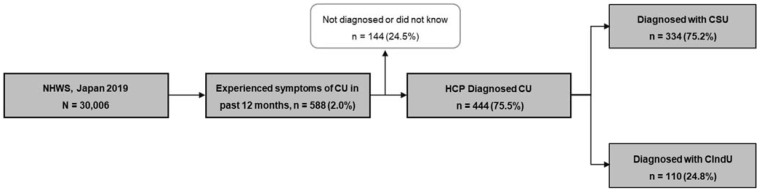
Patient flow: frequency of respondents with symptoms and diagnosed CSU. CIndU: chronic inducible urticaria; CSU: chronic spontaneous urticaria; CU: chronic urticaria; HCP: healthcare provider, NHWS: National Health and Wellness Survey.

A total of 30,006 respondents participated in the 2019 Japan NHWS, of whom 588 (2.0%) reported experiencing symptoms of CU (itch and hives) in the past year. Of those reporting CU symptoms, 75.5% (N = 444) received a physician’s diagnosis of CU, and among them, 75.2% (N = 334) had a CSU diagnosis, and 24.8% (N = 110) had a CIndU diagnosis (Figure 1). The overall 12-month weighted prevalence of diagnosed CSU in Japan was 1.1% (95% confidence interval: 1.1–1.1%).

#### 3.1.1. Sociodemographic and General Health Characteristics of Patients Diagnosed with CSU

Among patients with diagnosed CSU, 62.3% were female, and the mean (SD) age at data collection was 50.8 (15.3) years. Approximately two-thirds of patients had a university degree, 38.6% were employed full-time, and 59.9% had an annual income ranging from JPY 3,000,000 to < JPY 10,000,000. Nearly all patients had insurance coverage, with 48.5% reporting social insurance and 43.4% having national health insurance (Table 1).

**Figure 2:**
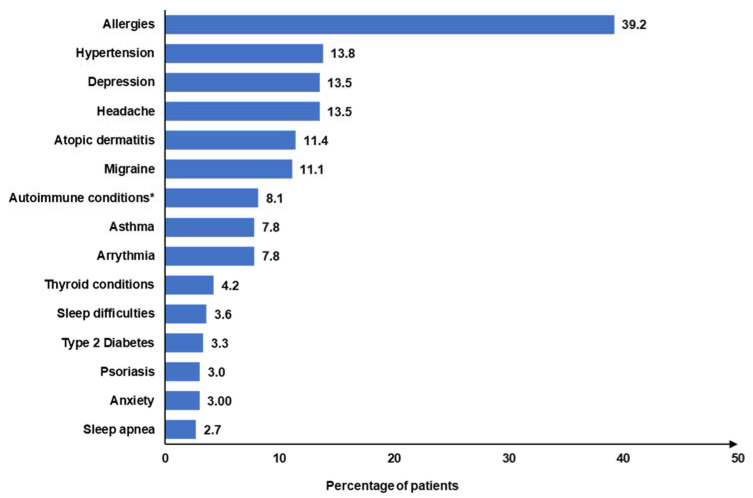
Frequently diagnosed comorbidities associated with CSU. CSU: chronic spontaneous urticaria. * Autoimmune conditions included the following diseases: rheumatoid arthritis (3.9%), ulcerative colitis (2.1%), Sjögren’s syndrome (0.9%), ankylosing spondylitis (0.6%), lupus (0.3%), and Crohn’s disease (0.3%).

The mean (SD) disease duration was 10.5 (12.4) years. In most cases, the diagnosis of CSU was confirmed by a dermatologist (71.8%), followed by a primary care physician (20.0%) and an allergist (7.7%) (Table 2). Only 3.3% of patients experienced angioedema in the 3 months prior to completing the NHWS, and the mean (SD) number of angioedema events among these patients in the past 3 months was 2.4 (2.7). The most frequently diagnosed comorbidities were allergies (39.2%), hypertension (13.8%), depression (13.5%), headache (13.5%), and atopic dermatitis (11.4%) (Figure 2). Most patients (88.0%) had a CCI score of 0, whereas 6.9% and 5.1% had a CCI score of 1 and 2+, respectively. More than half (61.1%) of the patients reported poorly controlled urticaria (UCT score <12), and only 36.8% of patients were being treated at the time of the survey. Among patients receiving treatment, H1-antihistamines (27.2%) were the most frequently prescribed medications, which included olopatadine (24.4%), fexofenadine (13.0%), and levocetirizine (12.2%), followed by bilastine (9.8%) and cetirizine hydrochloride (9.0%) (Table 2). Omalizumab was prescribed to only 0.8% of patients in this cohort.

#### 3.1.2. Humanistic Burden of CSU

**Figure 3:**
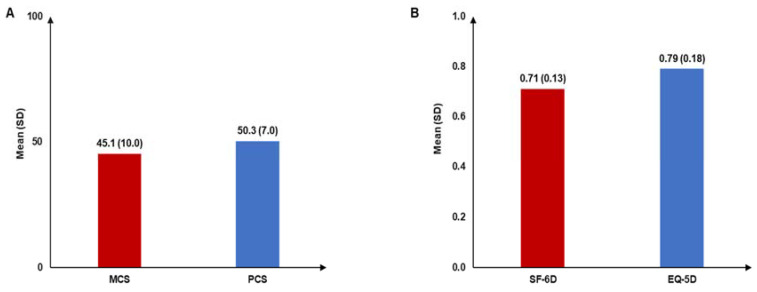
(**A**) SF-12 MCS and PCS scores; (**B**) health utility scores among patients with CSU. CSU: chronic spontaneous urticaria; EQ-5D: EuroQol 5-Dimension; MCS: mental component summary; PCS: physical component summary; SD: standard deviation; SF-12: Short-Form 12-Item Survey; SF-6D: Short-Form 6-Dimension. SF-12 MCS and PCS scores are reported as deviations from scores for the general population (i.e., 50 points); higher scores indicate better health status. SF-6D and EQ-5D scores range from 0 to 1; higher scores indicate better health status.

The mean (SD) SF-12 PCS and MCS scores were 50.3 (7.0) and 45.1 (10.0), respectively (Figure 3A), whereas the mean (SD) SF-6D and EQ-5D health utility scores were 0.71 (0.13) and 0.79 (0.18), respectively (Figure 3B). The mean (SD) EQ-VAS score was 68.06 (23.84).

**Figure 4:**
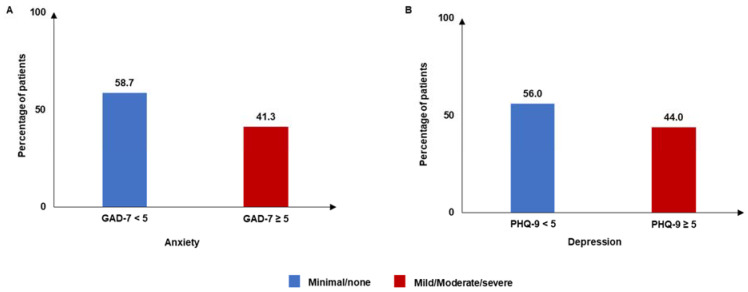
Mental status: (**A**) GAD-7; (**B**) PHQ-9 scores. GAD-7: General Anxiety Disorder-7; PHQ-9: Patient Health Questionnaire-9. GAD-7 < 5 and PHQ-9 < 5 scores indicate “none/minimal” anxiety and depression, respectively. GAD-7 ≥ 5 and PHQ-9 ≥ 5 scores indicate “mild/moderate/severe” anxiety and depression, respectively.

**Figure 5:**
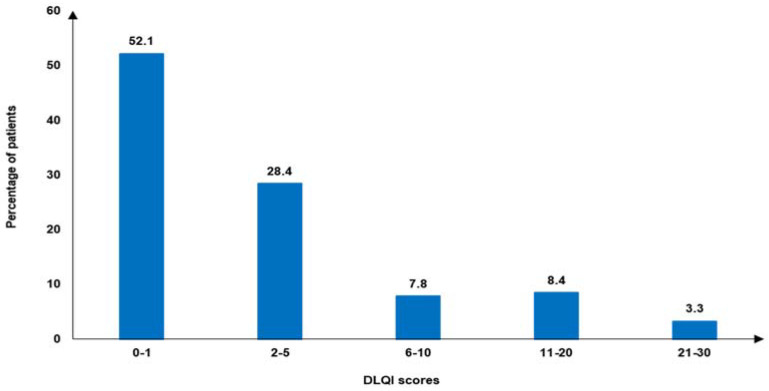
Categorized DLQI scores indicating effect on patients’ lives *. DLQI: Dermatology Life Quality Index. * 0–1: no effect; 2–5: small effect; 6–10: moderate effect; 11–20: very large effect; 21–30: extremely large effect.

In terms of mental health indicators, 41.3% of patients reported mild/moderate/severe anxiety (GAD-7 ≥ 5), while 44.0% reported mild/moderate/severe depression (PHQ-9 ≥ 5) (Figure 4). The mean (SD) DLQI score was 3.8 (6.0), and 11.7% of patients had a DLQI score >10. Based on the DLQI categories, more than half (52.1%) of patients reported a DLQI score of 0–1 (Figure 5).

#### 3.1.3. Economic Burden of CSU

**Figure 6:**
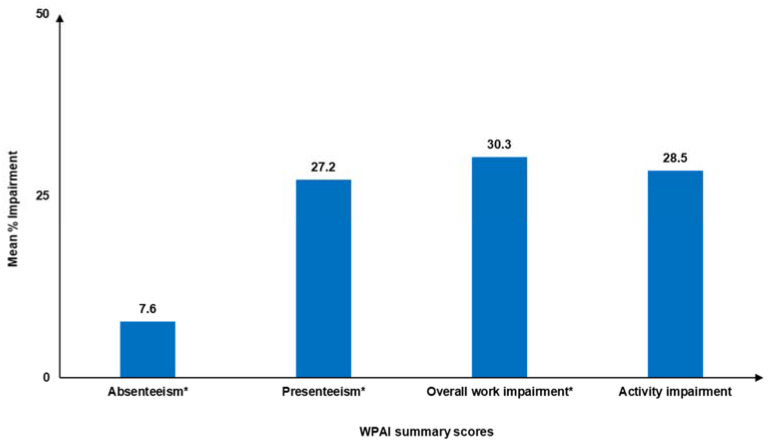
WPAI in the past 7 days. SD: standard deviation; WPAI: Work Productivity and Activity Impairment. * Assessed among employed respondents only. WPAI score ranges from 0% to 100%; higher scores reflect greater impairment of work and activity.

The mean % (SD) scores for absenteeism, presenteeism, overall work impairment, and activity impairment were 7.6 (17.6), 27.2 (27.2), 30.3 (29.6), and 28.5 (27.8), respectively (Figure 6).

**Figure 7:**
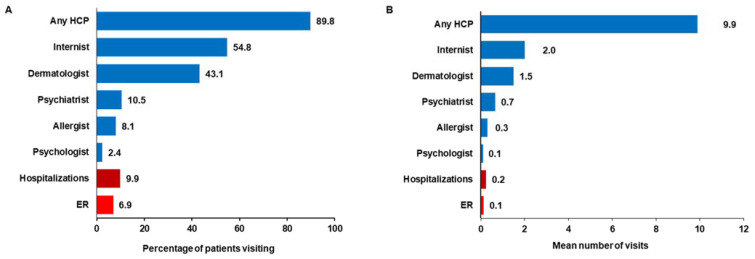
HRU among patients with CSU in the past 6 months: (**A**) percentage of patients with visits; (**B**) mean number of visits. CSU: chronic spontaneous urticaria; ER: emergency room; HCP: healthcare provider; HRU: healthcare resource utilization; SD: standard deviation. Note: Each proportion is represented as the number of patients who visited that specialist divided by the total number of patients with CSU.

Approximately 90.0% of patients reported visiting any HCP, 6.9% had ER visits, and 9.9% had hospitalizations in the past 6 months. Among HCPs, the most frequently visited specialties were internists (54.8%), followed by dermatologists (43.1%) and psychiatrists (10.5%) (Figure 7A). Over the past 6 months, the mean (SD) number of visits to any HCP, ER, and hospitalizations were 9.9 (11.8), 0.1 (0.7), and 0.2 (1.1), respectively (Figure 7B). The average monthly out-of-pocket costs ranged from JPY 1 to JPY 999 for 16.5%, JPY 1000 to JPY 4999 for 45.0%, and > JPY 5000 per month for 17.4% of patients.

## 4. Discussion

The existing published evidence on the prevalence and impact of CSU in Japan is limited, and the available studies tend to describe urticaria in general rather than focusing on CSU [13,14,15]. To our knowledge, this is the first study reporting a comprehensive evaluation of the prevalence and humanistic and economic burden of CSU using a nationally representative cohort in Japan.

The overall 12-month weighted prevalence of CSU in this study was estimated to be 1.1%, and CSU accounted for 75.2% of all physician-diagnosed CU. Our findings are aligned with those of other Japanese studies reporting 66.0–76.0% of urticaria cases being CSU [13]. The prevalence of CSU in the present study was higher compared with that in other Asian countries, such as South Korea (0.69–0.79%), but lower than that reported in China (2.7%) [5,7]. However, such a difference could arise from differences in the study design and cohorts assessed.

The mean age of patients diagnosed with CSU in this study was 50.8 years, and patients were predominantly female, which is consistent with findings from previous epidemiological studies in Japan [13,15]. The mean duration of CSU in this cohort was 10.5 years, highlighting the chronic nature of the condition and corresponding with the findings from another real-world study conducted in Japan (10.7 years for CU) [15]. In our study, the largest proportion of CSU cases were diagnosed by a dermatologist (71.8%), followed by primary care physicians (20.0%) and allergists (7.7%), reflecting the multidisciplinary approach often required for diagnosing and managing CSU.

In this study, the proportion of patients with CSU reporting angioedema was lower (3.3%) compared with that reported in previous studies in Japan (8.6–14.1%) [13,37] and studies outside Japan (33.0–67.0%) [2]. This discrepancy may be attributed to the poor perception of angioedema by the respondents as well as possible geographical and underlying pathological mechanisms.

Allergy, hypertension, depression, headache, and atopic dermatitis were the most common comorbidities in our cohort and were similar to the findings of other studies from Japan and South Korea [38,39]. More than half of the patients reported poorly controlled urticaria (UCT score <12), which was in line with a previous study in Japan [15]. Only one-third of patients were being treated at the time of the survey, indicating a treatment gap in CSU management, possibly due to inadequate awareness, limited access to specialists, or suboptimal treatment efficacy.

In addition to physical impact, CSU is associated with mental and emotional impairments [2,11]. The lower mean MCS score among patients with CSU versus that in the general population indicates a considerable impact on their mental well-being. The mean SF-6D and EQ-5D utility scores of 0.71 and 0.79, respectively, suggested that CSU also impacts patients’ overall health status. The impact of CSU on patients’ mental and overall health was comparable with that of atopic dermatitis in Japan [40]. More than 40.0% of patients in this cohort reported conditions of anxiety and depression, which corresponded with findings in other global studies [41,42,43]. The mean DLQI score of 3.8 indicated that CSU affected patients’ HRQoL to some extent, which was comparable to the finding from another study in Japan (mean DLQI score: 4.8) [15]. Although the DLQI score for patients with CSU was better than that for patients with other skin conditions (atopic dermatitis: 6.1 and psoriasis: 4.8), 11.7% of patients had a very large or extremely large effect on their QoL due to CSU [15]. Moreover, patients with CSU in this study reported impairment in overall work and activity, which is consistent with findings reported in other studies [15]. The majority of patients with CSU in this study reported visiting HCPs, with an average of 9.9 visits in the past 6 months. This result was also in line with that of a previous Japanese study reporting HCP visits among patients with CU, atopic dermatitis, and psoriasis [15]. This emphasizes the burden of medical consultations on patients as well as the healthcare system due to the chronic nature of CSU and the need for ongoing medical management.

### Study Limitations

Several limitations of this study should be noted. First, the NHWS is an internet-based survey. Although quota sampling was used to ensure demographic representativeness, there remains a possibility of selection bias owing to the population lacking internet access, potentially leading to an underestimation of the prevalence of CSU. Second, the NHWS is a self-administered questionnaire survey using patient-reported outcome measures with varying recall periods, which can introduce recall bias. Lastly, given that this is a cross-sectional study, the nature of the outcome data prevents any causal interpretation of the corresponding results.

## 5. Conclusions

In conclusion, this study provides robust and updated evidence on the prevalence of CSU in the Japanese population using nationally representative data. The study highlights the high prevalence of anxiety and depression among individuals with CSU, emphasizing the importance of addressing psychological well-being while also revealing the significant economic burden due to impaired work productivity and activity, as well as extensive healthcare resource utilization. The findings of this study also highlight the undertreatment of patients with CSU. By recognizing the prevalence and burden of CSU, healthcare providers can prioritize early diagnosis and treatment, comprehensive management strategies, and support for patients’ psychological well-being. This approach will not only improve patient outcomes and quality of life but also alleviate the economic burden associated with CSU.

## Figures and Tables

**Table 1:** Sociodemographic and general health characteristics of patients with diagnosed CSU (N = 334).

Sociodemographic and General Health Characteristics	
Age, mean (SD)	
At data collection	50.8 (15.3)
Sex, n (%)	
Female	208 (62.3)
Region, n (%)	
Hokkaido and Tohoku	33 (9.9)
Kanto	130 (38.9)
Chubu	56 (16.8)
Kinki	71 (21.3)
Chugoku and Shikoku	20 (6.0)
Kyushu and Okinawa	24 (7.2)
Education, n (%)	
Less than a university degree	106 (31.7)
University degree	223 (66.8)
Declined to answer	5 (1.5)
Employment, n (%)	
Employed full-time	129 (38.6)
Self-employed	31 (9.3)
Employed part-time	43 (12.9)
Homemaker	78 (23.4)
Not employed	32 (9.6)
Retired	13 (3.9)
Student	6 (1.8)
Short-/Long-term disability	2 (0.6)
Annual household income, n (%)	
Low: < JPY 3,000,000	51 (15.3)
Medium 1: JPY 3,000,000 to < JPY 6,000,000	119 (35.6)
Medium 2: JPY 6,000,000 to < JPY 10,000,000	81 (24.3)
High: JPY 10,000,000+	43 (12.9)
Declined to answer	40 (12.0)
Insurance type, n (%)	
National health insurance	145 (43.4)
Social insurance	162 (48.5)
Late-stage elderly insurance	13 (3.9)
Other	9 (2.7)
None of the above	5 (1.5)
Body mass index, n (%)	
Underweight (<18.5 kg/m^2^)	44 (13.2)
Normal weight (18.5 to <25.0 kg/m^2^)	218 (65.3)
Overweight (25 to <30.0 kg/m^2^)	45 (13.5)
Obese (>30.0 kg/m^2^)	9 (2.7)
Declined to answer	18 (5.4)

CSU: chronic spontaneous urticaria; SD: standard deviation; JPY: Japanese yen.

**Table 2:** Clinical characteristics of patients with diagnosed CSU (N = 334).

Age, mean (SD)	
At diagnosis	39.2 (14.9)
Diagnostician *, n (%)	
Primary Care Physician	44 (20.0)
Allergist	17 (7.7)
Dermatologist	158 (71.8)
Other	1 (0.5)
Charlson comorbidity index, n (%)	
0	294 (88.0)
1	23 (6.9)
2+	17 (5.1)
Urticaria control test, n (%)	
Poorly controlled (UCT < 12)	204 (61.1)
Angioedema in past 3 months	
Experienced angioedema, n (%)	11 (3.3)
Number of angioedema events, mean (SD)	2.4 (2.7)
Treatment patterns, n (%)	
Olopatadine	24.4
Fexofenadine	13.0
Levocetirizine	12.2
Bilastine	9.8
Cetirizine hydrochloride	9.0

CSU: chronic spontaneous urticaria; SD: standard deviation. * The proportion for diagnosticians is calculated from respondents who provided a valid response (N = 220).

## Data Availability

Data supporting this study are included within the article.
